# Minimizing the Spread of Negative Influence in SNIR Model by Contact Blocking

**DOI:** 10.3390/e24111623

**Published:** 2022-11-08

**Authors:** Caiyan Dai, Ling Chen, Kongfa Hu, Youwei Ding

**Affiliations:** 1 College of Artificial Intelligence and Information Technology, Nanjing University of Chinese Medicine, Nanjing 210023, China; 2 College of Information Engineering, Yangzhou University, Yangzhou 225012, China; 3 Jiangsu Collaborative Innovation Center of Traditional Chinese Medicine in Prevention and Treatment of Tumor, Nanjing 210023, China

**Keywords:** precise isolation, minimize virus infection, SNIR model

## Abstract

This paper presents a method to minimize the spread of negative influence on social networks by contact blocking. First, based on the infection-spreading process of COVID-19, the traditional susceptible, infectious, and recovered (SIR) propagation model is extended to the susceptible, non-symptomatic, infectious, and recovered (SNIR) model. Based on this model, we present a method to estimate the number of individuals infected by a virus at any given time. By calculating the reduction in the number of infected individuals after blocking contacts, the method selects the set of contacts to be blocked that can maximally reduce the affected range. The selection of contacts to be blocked is repeated until the number of isolated contacts that need to be blocked is reached or all infection sources are blocked. The experimental results on three real datasets and three synthetic datasets show that the algorithm obtains contact blockings that can achieve a larger reduction in the range of infection than other similar algorithms. This shows that the presented SNIR propagation model can more precisely reflect the diffusion and infection process of viruses in social networks, and can efficiently block virus infections.

## 1. Introduction

In recent years, there have been many infectious diseases in the international community which generally have the characteristics of rapid transmission and a wide range of harms. Therefore, scholars have studied the spread and control of epidemics [1] and proposed the SIR model. In this model, every individual is in one of three states: susceptibility, infection, or recovery. According to the characteristics of disease transmission, there are different improved SIR models. The specific information of the SIR model was described in [2]. Subsequently, a series of infectious diseases were studied based on the SIR model [3,4,5,6]. In this context, how to effectively control the spread of an epidemic is a research hotspot. One method to control the spread is to use the spectral norm of the minimized transfer matrix [7,8,9,10,11,12]. Another method is to use resource allocation [8] and improve the network model [11,12]. Some researchers have proposed studying the optimal control problem based on a given boundary [13,14] to minimize the cost of controlling the spread of an epidemic.

In the process of fighting COVID-19, isolating infected persons is an effective way to prevent infection. However, isolating every infected person (symptomatic or asymptomatic) comes with a certain price. In the case of large-scale infection, simply isolating all contacts of all infected persons would come with a great price. Therefore, we should adopt a strategy of precise isolation to block some essential contacts (i.e., edges in the network) among the people who have contact with the infected person, to minimize the final scope of virus infection at a reasonable cost.

Due to the openness and high speed of social networks, all types of false information and rumors can spread quickly and widely. Such false messages are typically accompanied by network hotspots or eye-catching information and tend to attract the attention of network users. False news will cause harm to individuals to some extent and can lead to social panic, a decline in business credibility, damage to personal reputation, and a serious loss of network security. Therefore, such misinformation must be controlled to make social networks more reliable and secure for information exchange. We must find efficient approaches to limit the destructive impact of negative influences.

Based on the infection process of COVID-19, we extended the traditional SIR model. The traditional model has the following limitations:

(1). The SIR model using traditional methods does not consider the state of asymptomatic infection. In the actual process of a COVID-19 infection, it has been proven that there is an asymptomatic phase; although if infected people are asymptomatic, they will infect others.
(2). The SIR model using traditional methods assumes that recovered people have antibodies, without considering the possibility of them becoming infected again. In the actual process of a COVID-19 infection, it has been proven that when antibodies are no longer present, recovered people will become susceptible and could be reinfected.
(3). In most traditional methods, only one transmission source, namely “patient zero”, is considered for a certain area. In the actual process of a COVID-19 infection, it has been proven that there may be multiple sources of infection in one region from various other regions.

Therefore, we propose the SNIR infection model. We studied the method of minimizing virus infection using the SNIR model based on the accurate isolation of multiple infection sources. At the same time, the model was also applied to describe the process of spreading rumors, in order to more accurately suppress them.

In recent years, methods have been proposed for the influence-blocking maximization problem using different propagation models. However, there are still many challenges in maximally blocking negative influences in complex social networks. The main challenges and difficulties are as follows:

(1). Most of the influence-blocking maximization algorithms assume that there is only a single source of negativity in the network. However, in real-world social networks, negativity is probably sent out from different channels that are linked with multiple sources. In order to block the influence from multiple propagation sources, we need to know the relationship between each node and each source. In addition, we also must consider the interactions between influences from different propagation sources, which is difficult to analyze due to the randomness of influence spreading.
(2). Most of the influence-blocking maximization algorithms try to limit the spread of negative influence by deleting some nodes, specifically by isolating some individuals in the social network. However, to prevent the spread of an epidemic, it is impossible to isolate individuals. A feasible way is to stop contact between some people.
(3). Some existing methods of influence-blocking leverage node centrality to select the nodes or edges to be blocked and ignore the propagation probability between nodes. Other methods use a BFS tree instead of the original network to simulate the influence propagation process. However, the BFS tree cannot precisely reflect the real process of influence propagation. In addition, these methods treat all nodes and edges in the network equally and ignore the latent topological features of the nodes. All of these factors reduce the quality of the results of influence-blocking.
(4). In most existing influence-blocking algorithms, a greedy strategy is used to select the nodes or edges to be blocked. In each step of greedy source node selection, a large number of simulations are needed to estimate the propagation range of the candidate source node set. Such a simulation of influence spreading is #P-complete, which requires large amounts of computation time; therefore, it is not applicable to large-scale networks. It is challenging to quickly and effectively find the contacts to be blocked in large-scale networks.

To tackle these difficulties, we propose the SNIR propagation model for influence spreading based on the spread of COVID-19 infections. Based on this model, we propose an algorithm named MaxExpectedH (maximum expected *H* values), in which the value of *H* changes with the spreading of the virus.

The main innovation and contributions of this paper are as follows:

(1). We propose the SNIR propagation model, which adds the asymptomatic infected state. The new model is more accurate than the traditional SIR model and can reflect the real propagation process of viral infection spreading.
(2). We propose a method for estimating the influence propagation range of each node at different times. Since we define a set of functions to estimate the probability for each node at different states at each time step, our method does not need to perform time-consuming simulations, thus it requires much less computation time than other methods.
(3). We present a method for selecting sets of contacts that need to be isolated in ascending order according to the value of the infection range of the virus.
(4). The experimental results show that the algorithm proposed in this paper can block negative influences more effectively than other methods. This shows that the presented SNIR propagation model can more precisely reflect the diffusion and infection process of viruses in social networks.

The remainder of this paper is structured as follows: A review of related works is presented in Section 2. In Section 3, the SNIR propagation model is proposed and the problem is defined. In Section 4, we propose a method to calculate the probability of nodes in different states. In Section 5, we propose an algorithm to calculate the range of influence spreading. In Section 6, an algorithm for selecting contacts to be blocked is proposed. Section 7 shows and analyzes the experimental results. Section 8 presents conclusions and further research.

## 2. Related Work

In recent years, by extending two traditional propagation models, the linear threshold (LT) model and the independent cascade (IC) model, many improved models have been proposed for analyzing maximum propagation in social networks [15].

Sahar et al. [16] proposed a path-based method for analyzing influence maximization in social networks. They noted that a small set of nodes, if activated, would spread information all over the network from two complementary perspectives, adapting the proposed algorithm to large-scale networks. Su et al. [17] proposed an algorithm for minimizing the seed set cost of influence spreading with a probabilistic guarantee. They define the problem as the minimum cost seed selection with probabilistic influence spreading guarantee in the linear threshold (LT) model. To avoid simulating the propagation of influence, an algorithm is adopted for estimating propagation by path counting in sample graphs. Masoud et al. [18] considered a new hybrid greedy approach based on a community detection algorithm and propose a MADM technique to cope with the optimization of influence when analyzing complex networks. They referenced community detection and the TOPSIS technique. Li et al. [19] proposed an alternative solution for the IM problem that attempts to select ordinary grassroots as seeds. First, they empirically proved that grassroots are a better choice than elites in the IM problem from the aspects of relationship strength and polarities, based on statistics and the analysis of real datasets. Then they developed a grassroots-oriented seed-users-seeking algorithm that fully explores the community information of the network structure. Calió et al. [20] proposed integrating a categorical-based notion of seed diversity into the objective function of a targeted influence maximization problem. They assumed that the users of a social network are associated with a categorical dataset, with each tuple expressing the profile of a user according to a predefined schema of categorical attributes.

In real social networks, there can be both positive and negative influences. Some researchers have focused on negative influences. The goal, through searching and setting negative influence sources, is to maximize the positive influence [21,22]. Kuhnle et al. [23] identified a new property, a generalized deterministic submodule, that ensures that propagation on the multiplex overall is submodular. In this case, they formulated an influential seed finder, a greedy algorithm with an approximation ratio (1-1/e). They also formulated an algorithm for knapsack seeding of the network that runs on each layer of the multiplex in parallel. Wang et al. [24] proposed the time-sensitive positive influence maximization problem by considering two factors simultaneously, to select the seed node set that would achieve the maximum spreading of positive influence within a specified time limit. Furthermore, they constructed a heat diffusion-based polarity influence diffusion model and an improved *k*-step greedy seed node selection algorithm to solve the TP-IM problem.

Some factors, such as vaccines and medical help, are also important in blocking the process of viral spreading. Khubchandani et al. [25] investigated the impact of COVID-19 morbidity and mortality among family and friends on vaccination preferences. They suggested that the dangers of not receiving the vaccine should be emphasized, as many people who do not know someone who was affected by COVID-19 are hesitant about vaccination. Long et al. [26] examined the spread of the COVID-19 pandemic in terms of social relationships. They specifically focused on the relational mechanisms of medical aid for people infected by COVID-19 and made recommendations for future public health policy and recovery.

At present, blocking negative influences is also a hotspot in the study of influence transmission. Song et al. [27] considered a more realistic situation, with the goal of reducing the number of rumor-infected users before a deadline, which they called the temporal influence blocking (TIB) problem. They proposed a two-phase solution called TIB-Solver to select k nodes to start a truth campaign such that the number of people reached by a rumor is minimized. Ghoshal et al. [28] leveraged the community structure of online social networks to select seed nodes statistically, independent of the distribution of misinformed nodes, for faster containment of misinformation with a simple one-time computation. They extended the work to include OSNs with the overlapped community as well. A competitive diffusion model was proposed for modeling the propagation of two types of competitive information in the same network [29]. The problem of minimizing the spread of rumors in social networks was explored and a novel heuristic based on diffusion dynamics was proposed to solve the rumor propagation problem under the LT1DT. The rumor propagation model can also be applied to the spread of infectious diseases.

Although the abovementioned methods are effective for the problem of blocking negative influences, their efficiency needs to be improved. Some of the algorithms select nodes or edges to be blocked based on their centrality and ignore the probability of propagation between nodes. Other methods simulate the process of influence spreading on trees instead of a general structured network. It is difficult for these methods to precisely detect the real diffusion sources without utilizing the latent structural information of the network. Furthermore, most of these approaches block the negative influence by isolating the nodes instead of the edges. This strategy is not practicable for preventing epidemics.

To overcome these defects, we propose a representation learning-based method for locating multiple influence sources. Compared with the other source-detecting methods, our algorithm has the following advantages:

(1). It is based on the SNIR propagation model, which enables us to learn more precisely about viral spreading. Therefore, the method can obtain more accurate results than the other methods.
(2). It establishes a set of functions to estimate the probability of each node in different states at each time step of influence propagation. Due to the nonparametric functions of the SNIR model, it is much easier to calculate probability with our method than with other methods.
(3). Based on the nodes’ probabilities in different states at a given time, our method precisely calculates the probability that each link will be blocked to prevent the spreading of negative influence. Since the model reflects influence propagation in the network, the influence probability obtained is more accurate than that estimated by Monto Carlo sampling.

## 3. Spreading Model and Problem Definition

In this section, we first propose the SNIR infection model, then give the problem definition.

### 3.1. SNIR Spreading Model

According to the actual process of COVID-19 infection, we propose the following SNIR infection model. In this model, every individual is in one of four states:

Susceptible (S): People in this state have not been infected, thus they will not infect others. They may become infected (turning to state I) with probability *β*, and they may also become infected but asymptomatic with probability *α* (turning to state N). Here, α+β<1.

Non-symptomatic (N): People in this state have been infected and will infect others, but they are asymptomatic. They will become infected with symptoms (turning to state I) with probability *δ*, and will recover with probability *η* and become convalescent (turning to state R). Here, δ+η<1.

Infectious (I): People in this state have been infected and have symptoms and will infect others. They will recover with probability *γ* and become convalescent (turning to state R). Here, γ<1.

Recovered (R): People in this state were infected, and now they have recovered. They have antibodies and will not infect others. However, without antibodies present, they will become susceptible (turning to state S) with probability *ξ*. Here, ξ<1.

**Figure 1:**
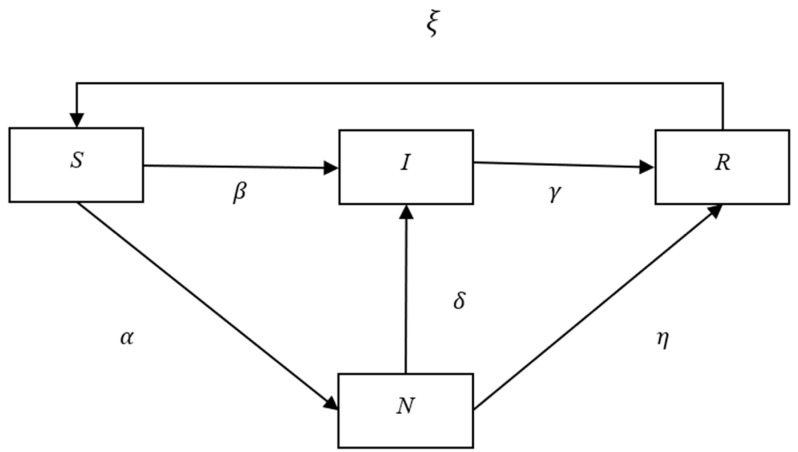
State transition process of the SNIR model.

The transformation relationship of the above four states is shown in Figure 1.

This model can also be used to describe the process of rumor propagation. Everyone’s state in the process of rumor propagation is as follows:

S: People in this state have not been exposed to rumors, thus they will not spread rumors to others. However, they will hear rumors with probability *β* and believe them, and then become believers (turning to state I). They will also hear rumors with probability *α* but have a neutral attitude about them (turning to state N).

N: People in this state have heard rumors and will spread them to others, but they have a neutral attitude about rumors themselves and do not fully believe them. They will become believers (turning to state I) with probability *δ*, and will reject rumors (turning to state R) with probability *η*.

I: People in this state have heard and believed rumors, and will spread them to others. They will also be influenced by positive information that changes their response to disbelief (turning to state R) with probability *γ.*

R: People in this state have accepted rumors in the past, but later, due to the influence of positive information, changed their ideas, became nonbelievers, and will not spread rumors to others. However, with changes in public opinion and the environment, over time they will also change to state S with probability ξ.

### 3.2. Setting the Transition Probabilities

In order to control the spread of an epidemic, people can be vaccinated to prevent infection, and after infected people recover, they may have antibodies to fight off the virus. All of these factors must be considered in the propagation model.

Let the protection rate of the vaccine be ω and the protection rate of antibodies be ψ. For people who have not been vaccinated and do not have antibodies, let the value of their probabilities α,β,δ,γ,η, and ξ be $\alpha^{\prime },\beta^{\prime },\delta^{\prime },\gamma^{\prime },\eta^{\prime },\;\mathrm{and}\;\xi^{\prime }$, respectively. Considering the effect of vaccines and antibodies, the transition probabilities can be set as follows:

$$\alpha =\left\{\begin{array}{cc} 1-\left(1-\alpha^{\prime }\right)\cdot \left(1-\omega \right) & \mathrm{if}\;\mathrm{vaccinated}\;\\ 1-\left(1-\alpha^{\prime }\right)\cdot \left(1-\psi \right) & \mathrm{if}\;\mathrm{antibodies}\;\mathrm{present}\\ \alpha^{\prime } & \mathrm{otherwise} \end{array}\right.$$

$$\beta =\left\{\begin{array}{cc} \beta^{\prime }\cdot \left(1-\omega \right) & \mathrm{if}\;\mathrm{vaccinated}\;\\ \beta^{\prime }\cdot \left(1-\psi \right) & \mathrm{if}\;\mathrm{antibodies}\;\mathrm{present}\\ \beta^{\prime } & \mathrm{otherwise} \end{array}\right.\;$$

$$\gamma =\left\{\begin{array}{cc} 1-\left(1-\gamma^{\prime }\right)\cdot \left(1-\omega \right) & \mathrm{if}\;\mathrm{vaccinated}\;\\ 1-\left(1-\gamma^{\prime }\right)\cdot \left(1-\psi \right) & \mathrm{if}\;\mathrm{antibodies}\;\mathrm{present}\\ \gamma^{\prime } & \mathrm{otherwise} \end{array}\right.$$

$$\delta =\left\{\begin{array}{cc} \delta^{\prime }\cdot \left(1-\omega \right) & \mathrm{if}\;\mathrm{vaccinated}\;\\ \delta^{\prime }\cdot \left(1-\psi \right) & \mathrm{if}\;\mathrm{antibodies}\;\mathrm{present}\\ \delta^{\prime } & \mathrm{otherwise} \end{array}\right.\;$$

$$\eta =\left\{\begin{array}{cc} 1-\left(1-\eta^{\prime }\right)\cdot \left(1-\omega \right) & \mathrm{if}\;\mathrm{vaccinated}\;\\ 1-\left(1-\eta^{\prime }\right)\cdot \left(1-\psi \right) & \mathrm{if}\;\mathrm{antibodies}\;\mathrm{present}\\ \eta^{\prime } & \mathrm{otherwise} \end{array}\right.$$

$$\xi =\left\{\begin{array}{cc} \xi^{\prime }\cdot \left(1-\omega \right) & \mathrm{if}\;\mathrm{vaccinated}\;\\ \xi^{\prime }\cdot \left(1-\psi \right) & \mathrm{if}\;\mathrm{antibodies}\;\mathrm{present}\\ \;\xi^{\prime } & \mathrm{otherwise} \end{array}\right.\;$$

### 3.3. Problem Definition

Given a social network *G* = (*V*, *E*, *P*), where *V* is the set of individuals, i.e., the network nodes, and *E* is the contact relationship between individuals, i.e., the network edges, probability *p_u,v_* on edge (*u*, v) represents the probability that the virus will be transmitted from *u* to *v*. Given the set of infected people observed *O =* $\left\{o_{1},\;o_{2,\;},\ldots ,o_{m\;}\;\right\}$, O⊂V, the *i*th observer $o_{i\;}\in O$ can be represented by a binary tuple $\left(o_{i\;},Q_{i}\right)$. Here, $Q_{i}\in \left\{N,I\;\right\}$, indicating $o_{i\;}$ in an infectious or non-symptomatic state. We assume that the cost of isolating a contact is 1, and a positive integer *k*, which is our predetermined cost, is given. Given the positive integer *T*, which is the maximum transmission time of the virus, we need to find out the contact edges *X* = {$e_{1},e_{2},\ldots ,e_{k}\}\;$ whose number is no more than *k*, so that in the graph *G* = (*V*, *E*\*X*, *P*), the range of virus infection is the smallest after time *T*. This supports the set of individuals in four states in the network at a certain time is *S*, *N*, *I*, *R*, and the range of virus infection at that time is |*N*|+|*I*|.

## 4. Calculation of the Probability of Nodes in Various States at a Given Time

Suppose the negative influence was instigated at time *t* = 0, and the probabilities of node *u* in states S,N,I, and R at time *t* are $p_{S}\left(u,t\right),p_{N}\left(u,t\right),p_{I}\left(u,t\right),\;\mathrm{and}\;p_{R}\left(u,t\right)$, respectively. Here, $p_{S}\left(u,t\right)+p_{N}\left(u,t\right)+p_{I}\left(u,t\right)+p_{R}\left(u,t\right)=1$.

Let $r\left(u,t\right)$ be the probability of virus transmission to *u* at time *t* and let *a*$\left(u,t\right)$ be the probability that *u* is infected by the virus at time *t*.

Set the initial value of the above variables (i.e., when *t* = 0) as:

(1) $$p_{S}\left(u,0\right)=\left\{\begin{array}{c} 1\\ 0 \end{array}\right.\begin{array}{c} \;u\in S\\ \;\mathrm{else} \end{array}p_{I}\left(u,0\right)=\left\{\begin{array}{c} 1\\ 0 \end{array}\right.\begin{array}{c} \;u\in I\\ \;\mathrm{else} \end{array}$$

(2) $$\;p_{R}\left(u,0\right)=\left\{\begin{array}{c} 1\\ 0 \end{array}\right.\begin{array}{c} \;u\in R\\ \;\mathrm{else} \end{array};\;p_{N}\left(u,0\right)=\left\{\begin{array}{c} 1\\ 0 \end{array}\right.\begin{array}{c} \;u\in N\\ \;\mathrm{else} \end{array}$$

(3) $$r\left(u,0\right)=\left\{\begin{array}{c} 1\\ 0 \end{array}\right.\begin{array}{c} \;u\in I\cup N\\ \;\mathrm{else} \end{array}\;a\left(u,0\right)=\left\{\begin{array}{c} 1\\ 0 \end{array}\right.\begin{array}{c} \;u\in I\cup N\\ \;\mathrm{else} \end{array}$$

Then, at each time *t*:

(4) $$r\left(u,t+1\right)=1-\underset{v\in \Gamma \left(u\right)}{\prod }\left[1-p_{v,u}\cdot a\left(u,t\right)\right]$$

Here, $\Gamma \left(u\right)=\left\{v|\left(v,u\right)\in E\right\}$ is the set of incoming neighbors of *u*, then

(5) $$a\left(u,t+1\right)=r\left(u,t+1\right).\left[\alpha +\beta \right]\cdot p_{S}\left(u,t\right)+\left(1-\gamma \cdot p_{I}\left(u,t+1\right)-\eta \cdot p_{N}\left(u,t\right)\right)\;$$

(6) $$p_{I}\left(u,t+1\right)=\left(1-\gamma \right)\cdot p_{I}\left(u,t\right)+r\left(u,t+1\right)\cdot \beta \cdot p_{S}\left(u,t\right)+\delta \cdot p_{N}\left(u,t\right)$$

(7) $$p_{R}\left(u,t+1\right)=\left(1-\xi \right)\cdot p_{R}\left(u,t\right)+\gamma \cdot p_{I}\left(u,t\right)+\eta \cdot p_{N}\left(u,t\right)$$

(8) $$p_{N}\left(u,t+1\right)=\left(1-\delta -\eta \right)\cdot p_{N}\left(u,t\right)+\alpha \cdot p_{S}\left(u,t\right)$$

(9) $$p_{S}\left(u,t+1\right)=1-p_{N}\left(u,t+1\right)-p_{I}\left(u,t+1\right)-p_{R}\left(u,t+1\right)$$

At each time, the individuals in the network W=N∪I are the source of infection. Let the time when the virus begins to spread be *t* = 0, and the probability that vertex *u* is in states S,N,I, and R at time *t* is $p_{S}^{w}\left(u,t\right),p_{N}^{w}\left(u,t\right),p_{I}^{w}\left(u,t\right),p_{R}^{w}\left(u,t\right)$. Then, the initial value can be set for each vertex *u* by Equations (1)–(3) as $p_{S}^{w}\left(u,0\right),p_{N}^{w}\left(u,0\right),p_{I}^{w}\left(u,0\right),p_{R}^{w}\left(u,0\right),r^{W}\left(u,0\right),\;a^{W}\left(u,0\right)$. Then, $p_{S}^{w}\left(u,t\right),p_{N}^{w}\left(u,t\right),p_{I}^{w}\left(u,t\right),p_{R}^{w}\left(u,t\right)$ can be obtained for each vertex *u* by iterative Formulas (4)–(9).

## 5. Calculating the Range of Influence Spreading

We first give an algorithm to calculate the range $I\left(G,\Omega \right)$ of virus infection at time T when the initial state is Ω = (*S*,*N*,*I*,*R*) on network G = (*V*, *E*, *P*). The algorithm first computes $P_{Q}^{v}\left(u,t\right)$ for all u∈V. When calculating the transmission process with W=N∪I as the infection source, because the virus only infects the neighbors related to vertex *v* in *W*, and not other individuals, it is not necessary to calculate the probability for all individuals, only the neighbors directly or indirectly related to *v*. After obtaining the probability $P_{Q}^{W}\left(u,t\right)$ (*u*∈V, *Q = S*,*N*,*I*,*R*, *t =* 1, 2, …, *T*), we know each vertex *u* with W=N∪I as the infectious source in state *Q* at time *t*. Later, the statistics of the following value are calculated:

(10) $$I\left(G,\Omega \right)=\overset{T}{\underset{t=0}{\sum }}\left[\underset{u\in V}{\sum }P_{N}^{W}\left(u,t\right)+\underset{u\in V}{\sum }P_{I}^{W}\left(u,t\right)\right]$$

We present an algorithm to estimate the expected value of influence spreading at each time *t*. The framework of the algorithm is given in Algorithm 1. The value of *H* is used to indicate the infection range of virus infection that changes with time *T* and it is related to the different state probabilities of each node. (V, E, P):(S, N, I, R):
**Algorithm 1:** Calculating the range of influence spreading. I (G, Ω)  **Input:** G = (*V*, *E*, *P*): Social network;   *p_u,v_*: Probability that the virus on edge (*u,v*) is transmitted from u to v;   Ω = (S, N,I,R): Set of individuals in four states in a network;   **Output:** I(G,Ω): Expected value of the range of virus infection up to time T;   **Begin**
  H = 0;   **For** each u∈V
**do**
  Initialize $p_{s}^{W}\left(u,0\right)\;\mathrm{and}\;\;p_{I}^{W}\left(u,0\right)$ based on (1);   Initialize $p_{N}^{W}\left(u,0\right)$ and $p_{R}^{W}\left(u,0\right)$ based on (2);   Initialize $r^{W}\left(u,0\right)$ and $a^{W}\left(u,0\right)$ based on (3); 
       
*H = H* + $\;p_{I}^{W}\left(u,0\right)+p_{N}^{W}\left(u,0\right)$;        **Endfor**
*u*;        *U* = N∪I;        **For**
*t* = 1 **to**
*T*
**do**
       *U*$\leftarrow U\cup \left\{\mathrm{neighbor}\;\mathrm{nodes}\;\mathrm{of}\;\mathrm{vertices}\;\mathrm{in}\;U\right\}$;   **For** each u∈U **do**
  Compute $r^{W}\left(u,t\right)$ and $a^{W}\left(u,t\right)$ based on (4) and (5);   Compute $p_{s}^{W}\left(u,t\right),\;\;p_{I}^{W}\left(u,t\right),$ $p_{N}^{W}\left(u,t\right)$ and $p_{R}^{W}\left(u,t\right)$ based on (6) to (9);       *H = H* + $\;p_{I}^{W}\left(u,t\right)+p_{N}^{W}\left(u,t\right)$;       **Endfor**
*u*;     **Endfor**
*t*;     $I\left(G,\Omega \right)=H;$
  **Output**($I\left(G,\Omega \right)$); **End**

Complexity analysis of the algorithm: Set the number of individuals in the network as *n*; the main calculation of the algorithm is the double cycle of *t* and *u*, and its complexity is O(*T*.*n*). Taking *T* as a constant, the complexity of the algorithm is O(*n*).

Compared with other methods based on epidemic spreading models, such as the SIR model, our method can block negative influence in a larger range in the network. Let *Ω_t_* = (*S*,*N*,*I*,*R*) be the set of states in the SNIR model at time *t*, and let *Ω*’*_t_* = (*S*,*I*,*R*) be the corresponding set of states in the SIR model. From (10) we can see that:

$$I\left(G,\Omega_{t}\right)=\overset{T}{\underset{t=0}{\sum }}\left[\underset{u\in V}{\sum }P_{N}^{W}\left(u,t\right)+\;\underset{u\in V}{\sum }P_{I}^{W}\left(u,t\right)\right]\;\geq \overset{T}{\underset{t=0}{\sum }}\underset{u\in V}{\sum }P_{I}^{W}\left(u,t\right)=I\left(G,\Omega \prime_{t}\right)$$

Therefore, the influence range estimated in our model is larger than that in SIR; consequently, the range of blocked negative influence is always larger.

## 6. Select Contacts to Be Blocked

The first infected individual should be the contact in W=N∪I. We determine the set of edges in contact with individuals in *W* as *Γ*(*W*)={*e*=(*u,v*)│*u*∈*W*, *v*∈*V*\*W*, (*u,v*)∈*E*}. For each edge *e* in $\Gamma \left(W\right)$, we constructed a graph $G_{e}$ after blocking *e*, then calculated the expected value $I\left(G_{e},\Omega \right)$ of the range of virus infections in $G_{e}$. We used the greedy method, taking edge *e* with the smallest expected value $I\left(G_{e},\Omega \right)$ step by step, and added set *X* of contacts that need to be isolated one by one until |*X|=k* or all contact edges in W=N∪I were blocked. The framework of the algorithm is given in Algorithm 2.
**Algorithm 2:** Identify the contacts to be blocked  **Input:**
*G* = (*V*, *E*, *P*): Interactive network;   *W*
=N∪I: Vertex set of infectious viruses in the network;     *k:* Threshold of isolation cost;   **Output:**
*X*: Set of contacts requiring isolation; **Begin**
  Construct the set of edges $\Gamma \left(W\right)=\left\{e=\left(u,v\right)|u\in W,\;\left(u,v\right)\in E\right\}$; **  ***j* = 0; *F* = E; *X* = Φ;
  **While**
*j < k* and $\Gamma \left(W\right)\neq \Phi$ **do**
  **For** each edge $e\in \Gamma \left(W\right)$ do **  **Construct graph $G_{e}=\left(V,\;F\backslash \left\{e\right\}\right)$; **  **$H\left(e\right)=I\left(G_{e},\Omega \right)$; /* Call algorithm 1 to calculate the expected value of the range of virus infection $I\left(G_{e},\Omega \right)$*/ **  Endfor** *e*; **  **Select edge *e* with the smallest $H\left(e\right)$ in $\Gamma \left(W\right)$; **  **$F=F\backslash \left\{e\right\}$; $X=X\cup \left\{e\right\}$
**  Endfor** *j*; **    output**(*X*); **End**


Complexity analysis of the algorithm: Suppose there are *m* infected persons, that is, $m=\left|N\cup I\right|$, and the maximum number of contacts of the infected person is *d_max_*, so we determine that the edges of contact with the individual in *W* do not exceed *m*.*d_max_*. The number of “while” loops in the algorithm is O(*k. m*.*d_max_*). Algorithm 1 is called in each loop, so the complexity of Algorithm 2 is O(*k. m*.*d_max_ n*). Taking *k*, *m,* and *d_max_* as constants, the complexity of the algorithm is O(*n*).

## 7. Experiments

### 7.1. Experimental Environment

The algorithm experiments were based on Windows 10, coded with Python 3.8, and run on an Intel^®^ Core^TM^ i7 CPU, 1.10 GHz. In order to verify the effectiveness of our proposed max expected *H* values (MaxExpectedH) algorithm, we tested it on multiple groups of real and synthetic networks, and compared its performance with the Random algorithm and MaxDegree algorithm [30].

### 7.2. Dataset and Parameter Setting

To verify the effectiveness of our MaxExpectedH algorithm, we tested it on four real networks and three synthetic networks with the other two algorithms. The three groups of real network data were dolphins [31], football [32], power [33], and Facebook [34]. Dolphins is a network that describes the family relationship of dolphins, in which each node represents a dolphin, and each edge indicates an association between two dolphins. Football is a network of American football, in which each node represents a college team participating in the 2000 football season, and edges connecting two nodes represent different matches between two teams. Power is a network of the topology of the power grid in the western United States, in which each node represents a power supply facility, and edges represent connections between power supply facilities.

Table 1 shows the main topological characteristics of the four actual networks, where *N* represents the number of nodes in the network, |*E*| represents the number of edges among the networks, and <*d*> represents the average degree of nodes in the network. The value of *H* is used to indicate the viral infection range, which changes with time *T,* and it is related to the different state probabilities of each node.

**Figure 2:**
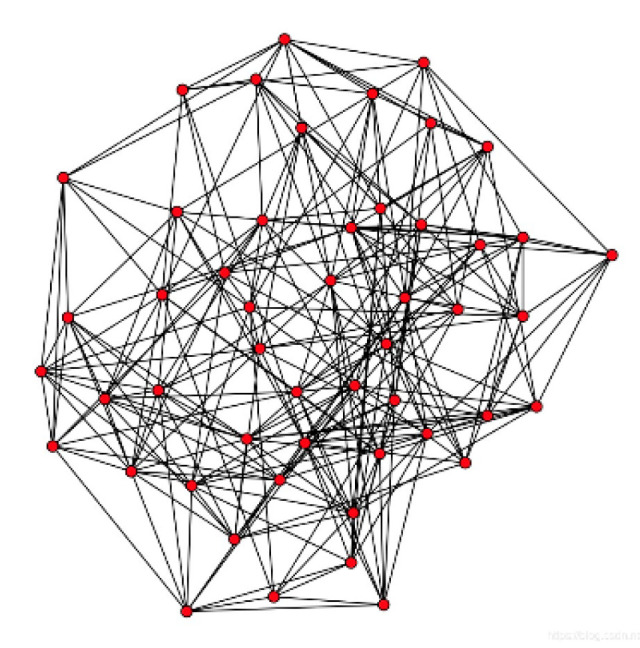
Small-world network.

**Figure 3:**
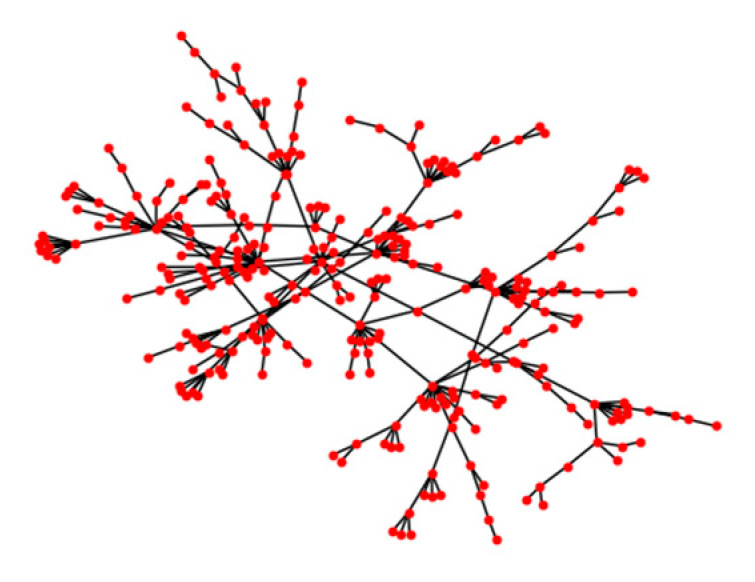
BA scale-free network.

The three synthetic networks are ER, WS small-world, and BA scale-free networks [34]. These are very close to real data, and the data of the ER and WS small-world networks are shown in Figure 2 and Figure 3.

The probability of S→N randomly takes $\alpha \epsilon \left[0.022,0.044\right]$, the probability of S→I randomly takes $\beta \epsilon \left[0.011,0.034\right]$, the probability of I→R randomly takes $\gamma \epsilon \left[0.28,0.35\right]$, the probability of N→I randomly takes $\delta \epsilon \left[0.012,0.031\right]$, and the probability of R→S randomly takes $\xi \epsilon \left[0.2,0.4\right]$. The experiments in the real and synthetic datasets are described below.

Table 2 shows the main topological characteristics of three synthetic networks, where *N* represents the number of nodes in the network, |*E*| represents the number of edges in different networks, and <*d*> represents the average degree of nodes in the network.

### 7.3. Experiments on Real Datasets and Analysis of Results

#### 7.3.1. Setting $p_{v,u}$ as a Fixed Value

Five nodes were randomly selected as the initially infected nodes, and their initial state values were set. Based on the average output of nodes, the probability $p_{v,u}$ of transmitting the virus from *v* to *u* was set as a value fixed to the opposite of the average degree of nodes in different networks.

**Figure 4:**
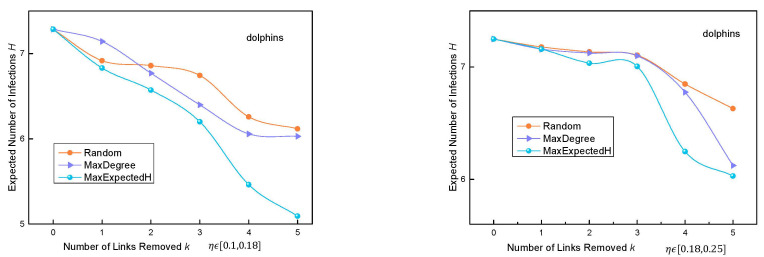

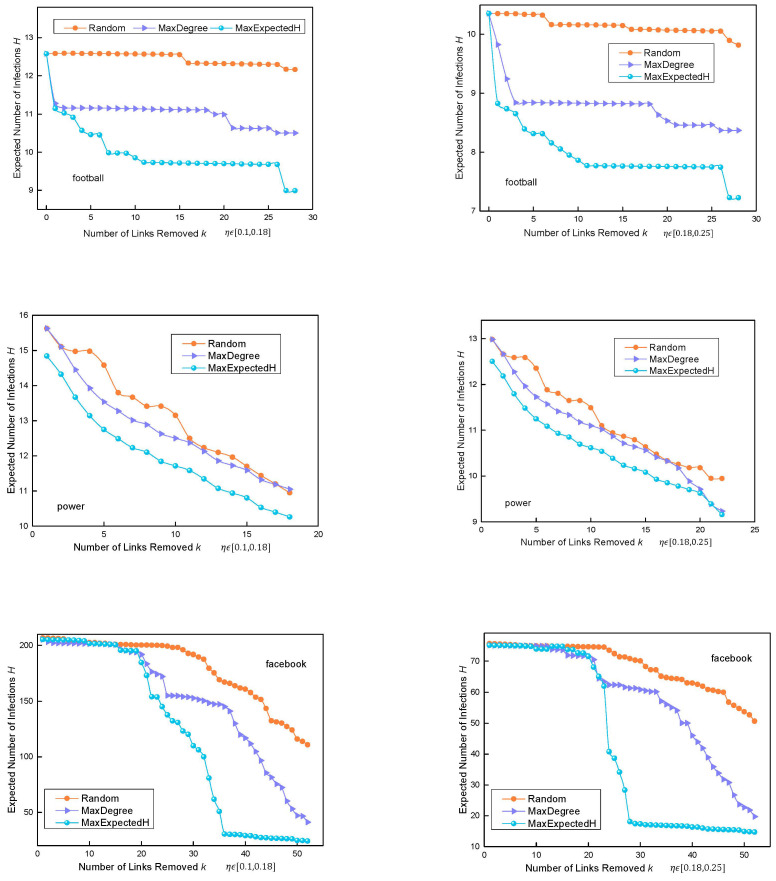
Change in H with change in k when $p_{v,u}$ is set to a fixed value in three real datasets.

In the four real networks, when the probability of N→R is randomly selected as $\eta \epsilon \left[0.1,0.18\right]$ and $\eta \epsilon \left[0.18,0.25\right]$, the operation of the three algorithms is as follows:

We tested the MaxExpectedH algorithm on four real networks. It can be seen from Figure 4 that when $p_{v,u}$ is set to a fixed value, *H* changes when k changes, and the change in *H* was compared with the Random algorithm and the MaxDegree algorithm. As can be seen from Figure 4, in the Football network, when fewer than five edges are removed, the superiority of the MaxExpectedH algorithm is not obvious. When more than five edges are removed, the *H* value of the MaxExpectedH algorithm is significantly less than the other two algorithms. In general, in these three real datasets, with an increase in the number of removed edges *k*, the performance of the MaxExpectedH algorithm is better than that of the other two algorithms.

#### 7.3.2. Setting $p_{v,u}$ as a Variable Value

**Figure 5:**
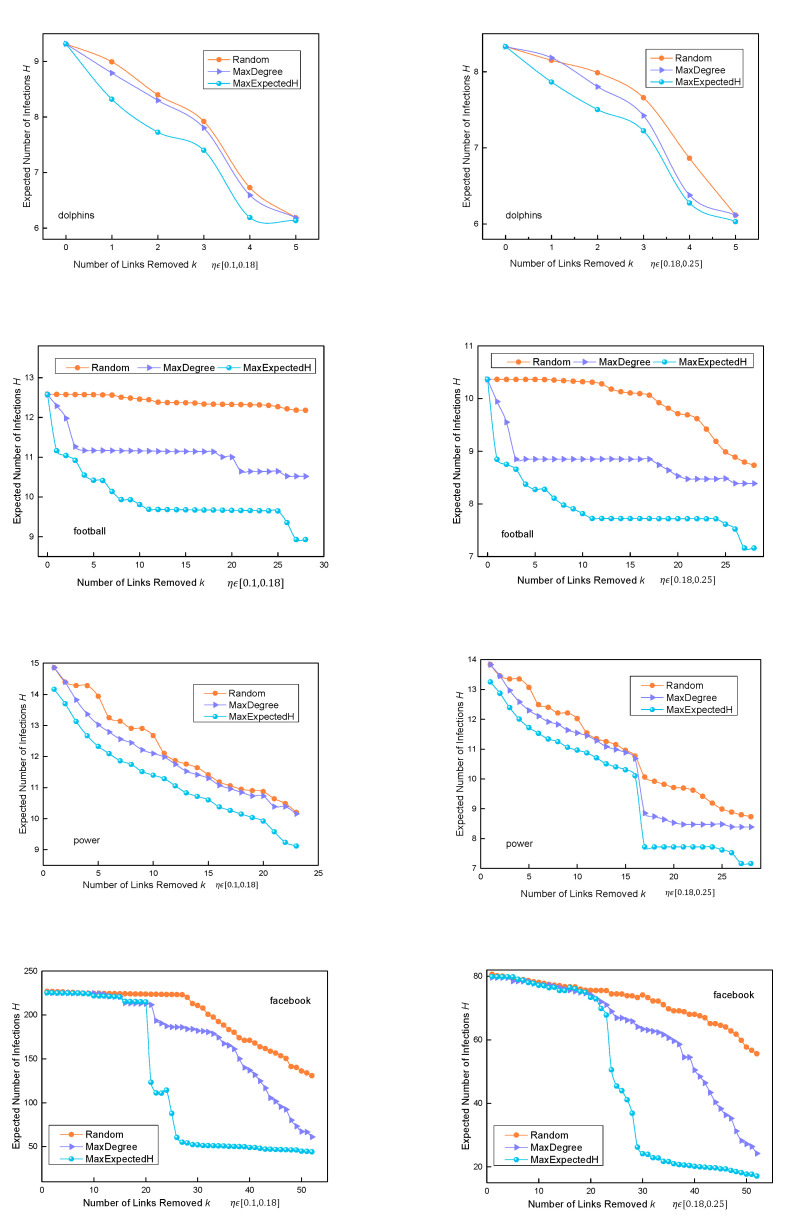
Change in *H* with change in *k* when $p_{v,u}$ is set to a variable value in three real datasets.

According to the different out-degrees of nodes, the probability $p_{v,u}$ of transmitting the virus from *v* to *u* was set to the reciprocal of the output of *v*. In the four real networks, when the probability of N→R is randomly selected as $\eta \epsilon \left[0.1,0.18\right]$ and $\eta \epsilon \left[0.18,0.25\right]$, the operation of the three algorithms is as follows:

We tested the accuracy of the MaxExpectedH algorithm on four real networks with $p_{v,u}$ set as a variable value. The change in *H* with the change in *k* can be seen in Figure 5. It can be seen in the figure that the MaxExpectedH is overall better than the other two algorithms in the Dolphins and Power networks. In the Football network, when fewer than five edges are removed, the *H* value of the MaxExpectedH algorithm is close to that of the MaxDegree algorithm. When more than five edges are removed, there is a wider gap between the two algorithms.

In the Facebook dataset, it can be seen that when fewer than 20 links are removed, the effect of our algorithm is not as good as that of the MaxDegree algorithm. This network link is tight. After removing an edge with the largest *H* value, the propagation of other edges is frequent and complex. However, when more than 20 edges are removed, the MaxExpectedH algorithm is superior to the other two models.

### 7.4. Experiments on Synthetic Datasets and Analysis of Results

The efficiency of the proposed algorithm was tested by generating three synthetic datasets: ER network, WS small-world network, and BA scale-free network.

The generated ER network contained 500 nodes, and the link probability was set to 0.025. The generated network *G*(*V*,*E*) contained 3153 edges. Five nodes were randomly selected as the initially infected nodes, and the state value of each node was set. The generated WS small-world network contained 500 nodes, and the generated network *G*(*V*,*E*) contained 2500 edges. Five nodes were randomly selected as the initially infected nodes, and the state value of each node was set. In the small-world network, the value of T was set to three. The generated BA scale-free network contained 300 nodes, and the generated network *G*(*V*,*E*) contained 300 edges. Three nodes were randomly selected as the initially infected nodes, and the state value of each node was set.

#### 7.4.1. Setting $p_{v,u}$ as a Fixed Value

**Figure 6:**
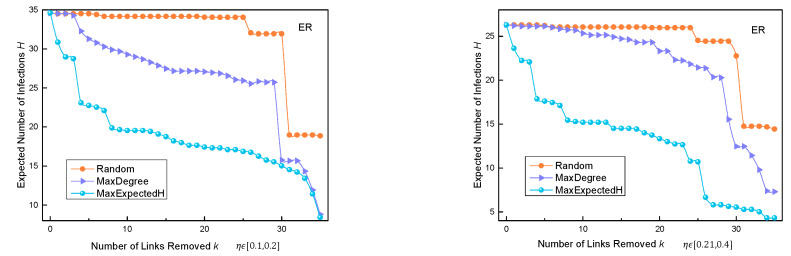

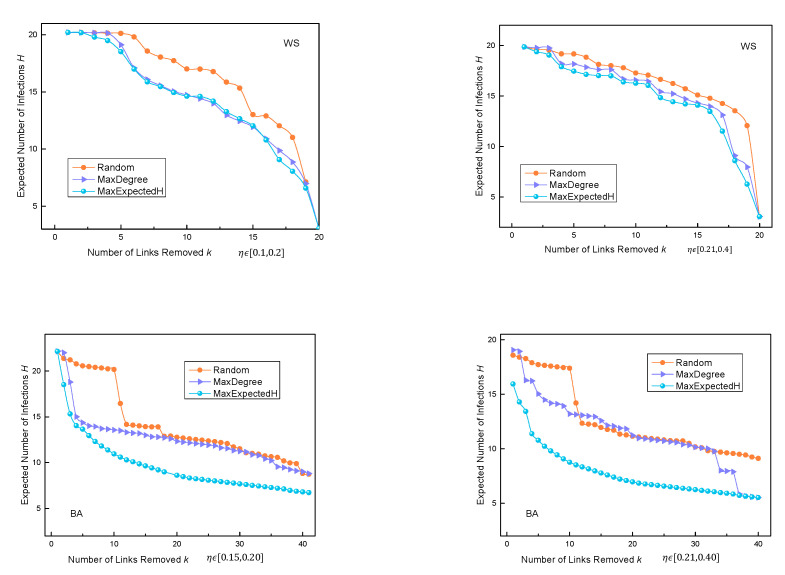
Change in *H* with change in *k* when $p_{v,u}$ is set as a fixed value in three synthetic datasets.

According to the average out-degree of the nodes, the probability $p_{v,u}$ of transmitting the virus from *v* to *u* was set as a value fixed to the opposite number of average degree of nodes in different networks. When the probability of N→R randomly choosing η in different ranges, the operation of the three algorithms is as follows:

We compared the three algorithms in the three synthetic networks. It can be seen in Figure 6 that, when $p_{v,u}$ is set to a fixed value, *H* changes when *k* changes, and the change in *H* was compared with the other two algorithms. The figure shows that the MaxExpectedH algorithm performed better than the other two algorithms in the synthetic ER and BA networks. Especially in the ER network, the MaxExpectedH algorithm had a lower *H* value than the other two algorithms when fewer edges were deleted. In the WS small-world network, the superiority of the MaxExpectedH algorithm was not obvious, because the average degree of nodes in this network was high. In the process of propagation, with an increase in deleted edges, there was still more propagation in the whole network. However, overall, the MaxExpectedH algorithm always achieved a lower *H* value than the Random and MaxDegree algorithms.

**Figure 7:**
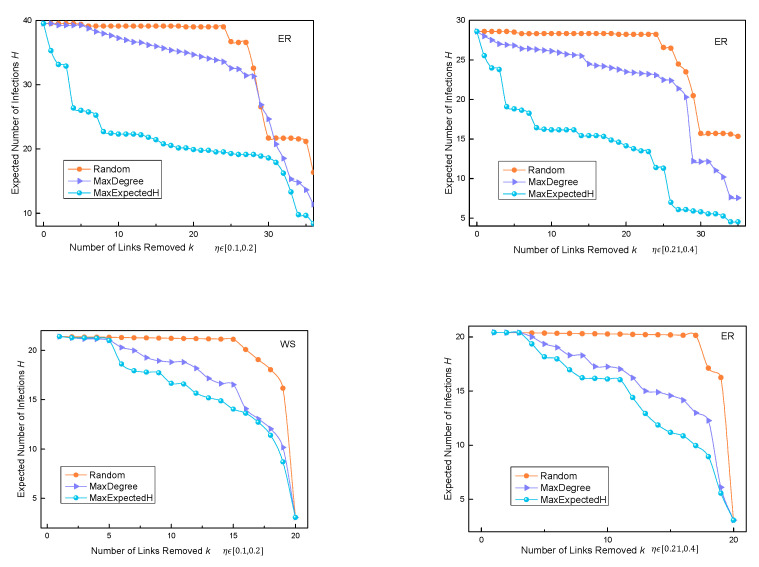

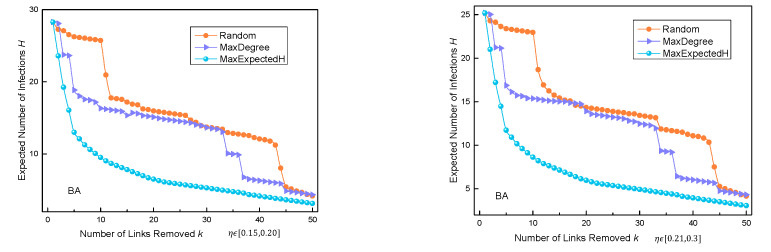
Change in *H* with change in *k* when $p_{v,u}$ is set as a variable value in three synthetic datasets.

It can be seen in Figure 7 that, when there are more initial nodes, the performance of our algorithm is better than that of the other two algorithms when the number of edges removed reaches about five. This shows that the performance of our algorithm is also ideal when the number of initially infected nodes in the ER network increases.

#### 7.4.2. Setting $p_{v,u}$ as a Variable Value

According to the different out-degrees of nodes, the probability $p_{v,u}$ of transmitting the virus from *v* to *u* was set to the reciprocal of the output of *v*. When the probability of N→R is randomly taken as η with different ranges, the operation of the three algorithms is as follows:

We compared three algorithms in three synthetic networks. It can be seen in Figure 6 that when $p_{v,u}$ is set as a variable value, *H* changes when *k* changes, and the change in *H* was compared with the other two algorithms. Figure 7 shows that the MaxExpectedH algorithm performs better than the other two algorithms in the synthetic ER and BA networks. Especially in the ER network, the MaxExpectedH algorithm had a lower *H* value than the other two algorithms when fewer edges were deleted. In the process of propagation, with an increase in deleted edges, there was still more propagation in the whole network. However, overall, the MaxExpectedH algorithm always achieved lower *H* values than the Random and MaxDegree algorithms.

In the WS small-world network, the *H* value of the MaxExpectedH algorithm was less than the other methods, but the difference was not significant. This is because the average degree of nodes in the WS network was high, and the superiority of MaxExpectedH is not obvious in a dense network.

## 8. Conclusions and Further Work

Based on the proposed SNIR model, in this paper, we mainly propose using the precise isolation method to minimize the spread of an epidemic. First, by combining the infectious characteristics of COVID-19 and the traditional SIR model, the SNIR model was proposed. Next, based on the established model, the infection range of the virus at different times was calculated according to its different state and diffusion probability. Then, the greedy method was used to select the set of contacts that need to be isolated until the preset value of the contact that needs to be isolated is reached or the infection source is completely blocked. In experiments on three real datasets and three simulated datasets, the results confirm the effectiveness of this algorithm in blocking virus infection.

One weak point of our method is that in a network with dense connections, the precision of our algorithm is not obviously higher than the others. Therefore, in further work, we will consider infected individuals and their adjacent edges as a whole, and the overall cost of isolating infected individuals and their adjacent edges together. The higher the isolation cost of infected individuals and their adjacent edges, the earlier they should be isolated so as to determine the sequence of isolated contacts. We will also consider the known propagation situation and subsequently locate the source of influence. We will look into finding the solution to the problem of maximizing influence as an optimization problem from the perspective of artificial intelligence and reduce the impact of randomness through the approach of linear regression in machine learning.

## Figures and Tables

**Table 1:** Four real networks.

Name	*N*	$\left|E\right|$	<*d*>
Dolphins	62	159	2.6
Football	115	613	5.3
Power	4941	6594	1.4
Facebook	4037	88,234	21.9

**Table 2:** Three synthetic networks.

Name	*N*	$\left|E\right|$	<*d*>
ER network	500	3153	6.3
WS small-world network	500	2500	5
BA scale-free network	300	300	1

## Data Availability

Publicly available datasets were analyzed in this study. This data can be found by the following links: https://download.csdn.net/download/u012311410/7670521, https://www.csdn.net/tags/NtzaIg5sMTczNzAtYmxvZwO0O0OO0O0O.html, accessed on 31 August 2022.

## Abbreviations

SNIR	Susceptible, non-symptomatic, infectious, and recovered
SIR	susceptible, infectious, and recovered
MaxExpectedH	maximum expected *H* value
